# Creativity in motion: examining the impact of meaningful movement on creative cognition

**DOI:** 10.3389/fcogn.2024.1386375

**Published:** 2024-08-26

**Authors:** Emily Frith, Stephanie E. Miller

**Affiliations:** ^1^Exponent, Inc., Bellevue, WA, United States; ^2^Department of Psychology, The University of Mississippi, University, MS, United States

**Keywords:** divergent thinking, embodiment, gesture, mind-body approach, movement

## Abstract

This study examined the link between creative cognition and meaningful physical movement among university students (*N* = 151) from a cognitive offloading perspective. A linear mixed model, repeated measures design examined whether divergent thinking on three Alternative Uses Tasks (AUTs; within-subjects factor) was influenced by movement in three independent experimental groups: (1) gesture as meaningful movement (*n* = 51), (2) meaningless movement (*n* = 50), and (3) restricted movement (*n* = 50). Linear mixed model results indicated that changes in AUT fluency, flexibility, and originality across trials did not significantly interact with group. However, baseline gesture frequency was positively associated with all four creativity outcome measures and moderated group-level changes in originality across trials. Infrequent baseline gesturers demonstrated lower originality scores in the gesture as meaningful movement group compared to those in the restricted movement group. More frequent baseline gesturers experienced higher originality in both the gesture as meaningful movement and meaningless movement groups compared to those in the restricted movement group. This study demonstrates that the cognitive processes involved in novel idea generation may be differentially impacted by movement and may be more sensitive to individual differences in movement predisposition than experimental movement manipulation.

## 1 Introduction

Given the broad impact of creativity on human experience, researchers have long been interested in examining the cognitive basis of creativity. Cognitive perspectives generally argue that creative thinking is the result of an effortful problem-solving process, during which ordinary mental abilities work together to yield extraordinary, novel, and/or unexpected ideas and solutions (Weisberg, 1993; Sternberg, 1999). However, we still know relatively little about how creative cognition operates in a larger context. The embodied cognition approach suggests that thinking is a function of meaningful relationships between the mind and body (Wilson, 2002; Glǎveanu, 2013). Less research has explored the ways in which creative divergent thinking may be impacted by these dynamic mind-body relationships (see Frith et al., 2019). Therefore, the purpose of this study was to examine how movement may impact divergent thinking, with a specific emphasis on how task-relevant movement may aid the effortful cognitive processes involved in generating novel and unique responses.

Laboratory studies often measure divergent thinking as one approach for assessing creative cognition (Guilford, 1950, 1967, 1988; Runco and Acar, 2012). Divergent thinking assessments require individuals to generate many ideas from a single prompt, such as brainstorming unique and useful ways to solve a problem (Guilford, 1967). Crucially, individuals must try to avoid providing conventional ideas and instead offer unconventional responses. The Alternative Uses Task (AUT) is one of the most common measures of divergent thinking because it is thought to yield three distinct, measurable factors that are expected to sufficiently capture individual variability in creative thoughts and behaviors: (1) fluency (i.e., the ability to generate many ideas from a single stimulus), (2) flexibility (i.e., the ability to generate ideas that span diverse categories), and (3) originality [i.e., the ability to produce unique solutions (Guilford, 1967; Silvia et al., 2009)]. Additionally, divergent thinking tests have been widely employed in laboratory environments and have been shown to demonstrate modest predictive validity for creative thinking in real-world settings (Runco and Acar, 2012; Beaty et al., 2013; Jauk et al., 2014; Adnan et al., 2019).

Although it is challenging to account for various factors, such as development, experience, and social context, which may influence creative ability outside of controlled laboratory settings (Barron and Harrington, 1981; Runco, 2008; Runco and Acar, 2012), the cognitive perspective suggests that divergent thinking assessments are useful for detecting creative potential and studying the fundamental cognitive processes involved during creative thinking and problem-solving (Ward et al., 1999; Zeng et al., 2011; Runco and Acar, 2012). In addition, it is reasonable to expect that even across individual factors and contexts, those same fundamental cognitive processes are needed to think creatively (Ward et al., 1999). Although there are inherent limitations in creativity assessment, divergent thinking measurement incorporates several empirical strengths, which is why the AUT remains a primary method for examining creative potential and the cognitive processes involved (Plucker and Renzulli, 1999; Silvia et al., 2008). AUT performance is contingent not only on access to, and manipulation of, existing knowledge representations (Silvia et al., 2008), but also emphasizes individuals' ability to generate multiple, original responses to stimuli that are inherently unexpected and vary across individuals (Cropley, 2006). Variability across the core elements of divergent thinking: fluency, flexibility, and originality, offers substantial measurement value as individual differences can be examined with the AUT (Silvia et al., 2008). This is important for extending work in creative cognition because variation in individual performances may offer a more fine-grained analysis of how creative cognitions operate in response to experimental manipulations (Kaufman et al., 2008; Beaty et al., 2014).

The measurement of divergent thinking from a creative cognition perspective has led researchers to a better understanding of the mental factors that impact creative thinking and problem-solving. However, our understanding of creative cognition may not be complete without an investigation of movement within a broader embodied context (Wilson, 2002; Wilson and Golonka, 2013), as work has demonstrated that movement and thinking often interact as people navigate the problem-solving environment (Wilson, 2002; Barsalou, 2008; Thomas and Lleras, 2009). This interplay has more specifically been examined within embodied cognition, where *embodiment* is often defined as task-relevant physical movement that can be effectively used to stimulate, direct, or preserve mental resources during problem-solving, and is thought to be heavily influenced by contextual factors (e.g., presence of other individuals, task demand, etc., Wilson, 2002; Tversky, 2009; Wilson and Golonka, 2013).

Embodied creativity research suggests that person-context relationships underpin creative thinking; that is, tools, materials, technologies, and environments in which people operate are fundamental to creative thought and behavior, and bodily experiences are critical for creating meaning and expressing thought (Malinin, 2019; Griffith, 2021; Richard et al., 2021). Indeed, several studies indicate that physical movement in combination with thought (e.g., environmental exploration, roaming, and physical manipulation, among others; see Kimmel and Groth, 2023) may offer a greater boon to creative cognition than thought alone. For example, Hao et al. (2015) demonstrated that when participants were permitted to write down their ideas during a divergent thinking task, they generated more ideas than participants asked to report their ideas aloud, relying on cognitive processes alone. The authors proposed that this is because verbal reports are more cognitively demanding than writing. A complimentary explanation worth considering is that moving a pen across paper may help offload cognitive effort onto the environment, thereby reducing cognitive demand and allowing for effort allocation toward idea generation (Risko and Gilbert, 2016). Relatedly, several researchers have shown that fluid movement (i.e., roaming freely in a laboratory space) during effortful brainstorming influences the generation of original ideas (Leung et al., 2012; Kuo and Yeh, 2016; Zhou et al., 2017), and that physical improvisation, role-play, and creative drama favorably influence creative thinking and suggest that the body is an important part of creative expression (Byrge and Tang, 2015; Richard et al., 2021; Torrents et al., 2021). Taken together, these examples suggest that engaging in task-relevant movement may favorably impact the effortful cognitive processes involved in creative thought. This is because, as task-relevant movement is posited to be functionally similar to thought, altering one's environment through embodiment may help reduce cognitive load by making mental operations quicker, simpler, and/or more reliable during creative thinking (Kirsh and Maglio, 1994).

Embodied cognition perspectives argue that movement and thought interact in a bidirectional relationship such that movement supports effortful problem-solving, and thought processes often elicit movement (Wilson, 2002; Thomas and Lleras, 2009; Tversky, 2009). Aligned with this approach, it is reasonable to expect that task-relevant movement adds meaning to the mental workspace, which may then guide goal-directed representations and dictate the novelty and usefulness of creative responses. Therefore, we chose co-speech hand gestures as a form of meaningful movement manipulation in the present experiment for several reasons. First, gesture is defined as overt bodily movement that meaningfully conveys ideas and mental states (Goldin-Meadow, 2005). This is because gesture, specifically, may serve as a tool that is functionally similar to cognitive processes, such as using hand motions to signify language (e.g., a basketball coach giving his athletes the thumbs-up signal after a play, or friends waving to one another across a crowded restaurant; Tversky, 2009). In fact, a wealth of embodied cognition research has focused on the potential for gesture to aid effortful problem-solving (Feyereisen and de Lannoy, 1991; McNeill, 1992; Garber and Goldin-Meadow, 2002; Goldin-Meadow and Beilock, 2010; Cook et al., 2012; Marstaller and Burianová, 2013). Second, research has demonstrated benefits of gesture for both explaining and learning novel concepts (Broaders et al., 2007; Cook et al., 2008; Goldin-Meadow, 2010). This may suggest that gestures are not only reflective of cognitive processes but play a role in creating them (Goldin-Meadow, 2010). This also highlights the body's role in constructing meaningful mental representations. Third, the efficacy of gesture as meaningful movement has been compared to movement that is incompatible with effortful mental representations, such as producing repetitive circular hand motions while explaining math (Cook et al., 2012). Task-irrelevant movement is incompatible with the idea of meaningfully altering one's environment using movement to accomplish task goals (Clark and Chalmers, 1998; Cook et al., 2012). Task-irrelevant, contextually meaningless movements divert attention from the target task, amplify cognitive load, and have been shown to impair problem-solving performance (Cook et al., 2012). Similarly, preventing gestures has also been associated with disruptions in lexical access that parallel the effects of increasing cognitive load. For example, participants instructed to refrain from gesturing while generating narrations of cartoons, or from providing narrations that contained the letters “c” or “d” under increased working memory load, experienced similar lexical errors, including verbal disfluency and nonproductive pauses in speech, which offers additional support for the potential role of co-speech gesture in cognitive offloading (Rauscher et al., 1996).

Thus far, work suggests that embodiment is capable of favorably impacting creative problem-solving, but that meaningful movement may be particularly beneficial. One theory within the embodiment literature that aligns with existing evidence is the cognitive offloading theory (COT). The COT proposes that meaningful movement is capable of attenuating cognitive effort and preserving executive functioning because the mind is less constrained by internal processing demands (Kirsh and Maglio, 1994; Scaife and Rogers, 1996; Wilson, 2002; Martin and Schwartz, 2005; Pouw et al., 2014; Risko et al., 2014; Risko and Gilbert, 2016). To illustrate, Cook et al. (2012) asked college-aged participants to first solve a mathematical equation using pen and paper. Afterwards, they were shown six letters on a computer screen and were instructed to remember them while verbally explaining how they solved the initial math problem. Participants explained their solutions while either producing co-speech gestures, meaningless movement (i.e., making repetitive circular motions with the hands), or no movement. Gesturing was associated with higher letter recall than moving one's hands in meaningless circles and not moving at all. This research demonstrated that meaningful gestures may reduce cognitive load, thereby preserving task-relevant working memory resources, because co-speech gestures are a conduit for the expression of meaning (Cook et al., 2012). However, the COT has primarily been assessed in the context of standard problem-solving assessments (Goldin-Meadow et al., 2001; Wilson, 2002; Cook et al., 2012; Risko et al., 2014; Risko and Gilbert, 2016). Applying the COT to creative cognition is a natural extension, as fundamental cognitive abilities (e.g., memory and executive function) involved in standard problem-solving are integral to creative thinking and problem-solving as well (Ward et al., 1999).

Given that creative cognition may be best understood by using meaningful physical movement as a way to offload mental work and preserve task-specific executive resources, we sought to examine whether meaningful, task-relevant movement influences effortful creative divergent thinking under three movement manipulation conditions. We assigned participants to either a meaningful gesture, task-irrelevant (i.e., meaningless) movement, or restricted movement manipulation while responding to a divergent creativity task (the AUT). We also used a within-subject AUT trial structure (i.e., baseline, movement manipulation, and movement manipulation + cognitive load) to investigate the effects of such movement manipulations relative to participants' baseline AUT performance and under conditions of cognitive load, given that gesture was hypothesized to reduce cognitive load. We also explored the role of individual differences in baseline gesture on divergent thinking performance.

## 2 Materials and methods

Undergraduate and graduate student participants at the University of Mississippi (*n* = 151) were recruited for a one-visit experiment (see Table 1 for descriptive statistics of the sample). The sample size determined for this project was estimated following an a-priori power analysis, considering data from Kirk and Lewis (2017) who found a correlation between gesture production and creative fluency in the AUT, along with a significant increase in the generation of creative ideas when participants were encouraged to gesture in the AUT. We used inputs of *f* = 0.12 (Cohen's *d* = 0.23), alpha = 0.05, and power of 0.80. A linear mixed model, repeated measures design examined the effects of a movement manipulation (between-subjects factor) on changes in divergent thinking performance [i.e., fluency, flexibility, originality (rarity), and originality (subjective) across the AUT trial structure (within-subject factor)].

**Table 1:** Descriptive statistics of the sample.

**Variable**	**Point estimate**	**SE**	**Range**
Age	18.97	0.157	18–33
Gender (% female)	64.7	–	–
GPA	3.47	0.039	1.8–4.0
Handedness (% right)	86.8	–	–
Hours slept last night	6.88	0.136	3–13
Hunger (average rating on a 1–5 Likert scale)	2.08	0.098	1–5
Race-Ethnicity (% Caucasian)	76.2	–	–

GPA, grade-point average.

### 2.1 Procedure

**Figure 1:**
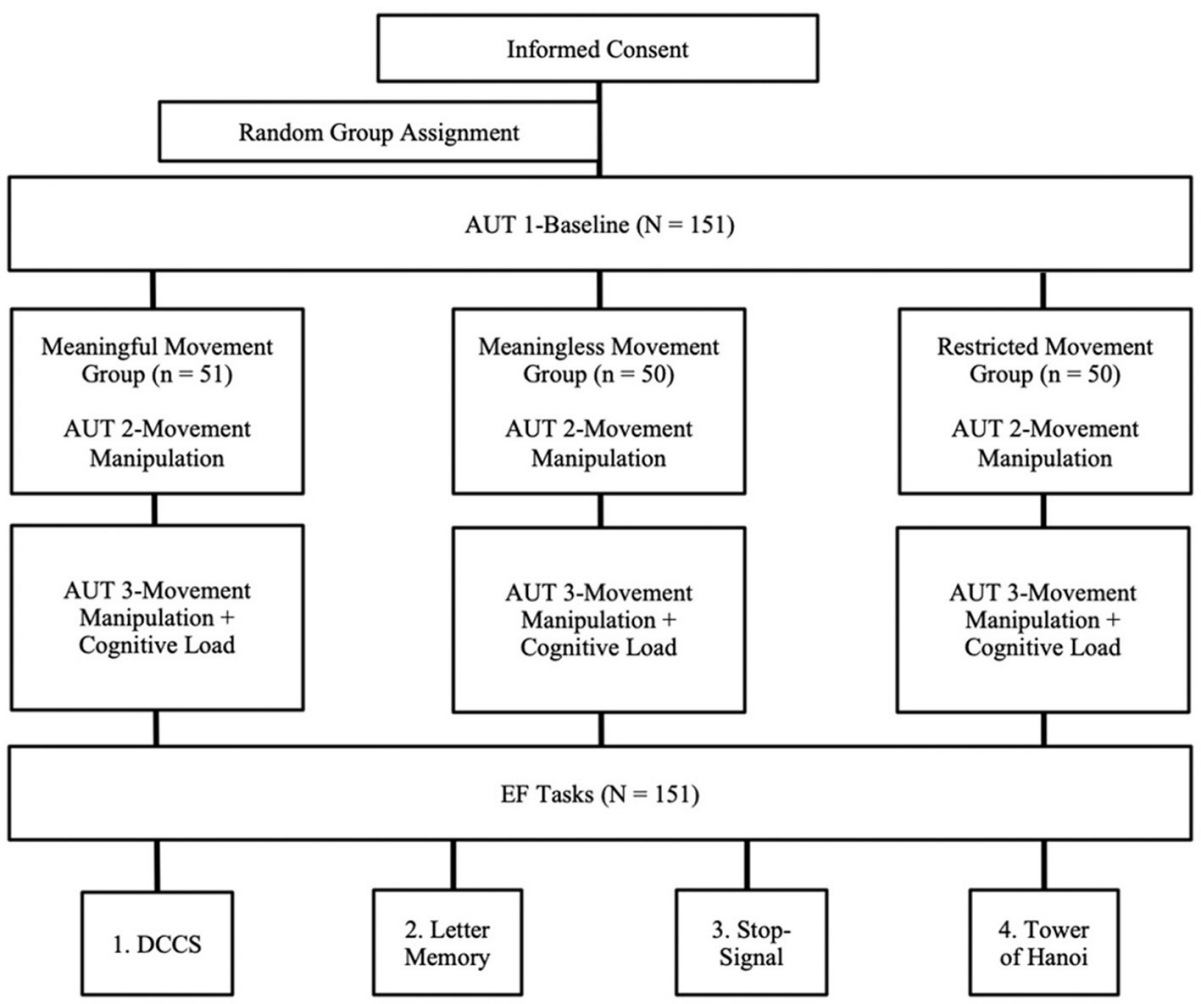
General experimental procedures. AUT, Alternative Uses Task; DCCS, dimensional change card sort; EF, executive function.

One of four research assistants tested participants individually, in a quiet room free of distractions. Participants first provided written informed consent to (1) participate in the research study and (2) to allow a portion of the study to be video-recorded (to facilitate gesture coding). Next, participants supplied demographic information (see Table 1), and then were randomly assigned to one of three independent, embodied manipulation groups: (1) meaningful movement (*n* = 51), (2) meaningless movement (*n* = 50), and (3) restricted movement (*n* = 50). Each participant completed three AUT trials (i.e., baseline, movement manipulation, and movement + cognitive load). At the end of the visit, all participants completed four executive functioning measures in the same order. These measures are not included as part of the current analyses (see Figure 1 for experimental procedures).

### 2.2 Divergent thinking assessments (AUTs)

Each participant completed three AUTs, as AUT trial structure represented the within-subject factor. For each trial, experimenters transcribed all AUT responses, and participants were video-recorded to facilitate gesture coding. The stimulus items pencil, shoe, and key were selected after reviewing previous research that matched these items according to both the number of actions that may be performed on a stimulus (Guérard et al., 2015), and ease of pantomiming, which was defined as a feature that may influence the likelihood of people expressing meaning through hand gestures (Guérard et al., 2015; Kirk and Lewis, 2017). Amenability to action and pantomiming metrics were important to AUT selection for this experiment because we wanted to ensure that the three stimuli were compatible in how easily they could be (1) physically acted upon or manipulated in a variety of ways and (2) physically used to communicate ideas via gesture. We also counterbalanced each AUT stimulus.

#### 2.2.1 Baseline AUT 1

For the first AUT, the experimenter encouraged each participant to be creative, as previous work indicates that such instructions clarify task expectations, reinforce goal-directed effort, and guide the cognitive process of creativity (Niu and Liu, 2009; Beaty and Silvia, 2012; Forthmann et al., 2016). Participants were verbally presented with the following general task instructions for the baseline assessment:

“In this task, I would like you to come up with as many uses for [a _____] as possible. You will have three minutes to do so. Please be creative, and come up with ideas that are clever, humorous, original, or interesting. Remember to name as many alternative uses for [a ______] as you can and please be as creative as you can while I write down your responses.”

Prior to providing responses for each trial, participants were asked if they understood. The experimenter explained the task again if the participant did not understand and reminded all participants that they would have 3 min to think of alternative uses for the item.

#### 2.2.2 Movement manipulation AUT 2

As aforementioned, participants were randomly assigned into one of three independent, movement manipulation groups (i.e., meaningful gesture, meaningless, and restricted movement groups). After the 3-min baseline task concluded, participants were again read the same initial instructions for the baseline assessment, but were also told:

“Sometimes people are more creative if they focus carefully on their ideas. One way to increase focus is to [use your hands (meaningful and meaningless movement groups)/remain still (restricted movement group] while explaining your responses. We would like you to do this. Please be sure to [gesture with your hands (meaningful group)/move your hands in circles (meaningless movement group)/remain as still as possible (restricted movement group)] while thinking of ideas for alternative uses.”

In the meaningful movement group, if the participant was not gesturing as initially instructed, the experimenter reminded them to please be sure to continue gesturing with their hands. In the meaningless movement group, the experimenter physically demonstrated how participants should move their hands in circles before the task began. In the restricted movement group, participants were first instructed to place their hands on the table in front of them, palms down, and were reminded by the experimenter to please refrain from gesturing if they used their hands during the task.

#### 2.2.3 Movement manipulation + cognitive load AUT 3

After 3 min had elapsed, participants were again read the baseline instructions and the movement manipulation instructions, but prior to beginning AUT 3 were told to “Please refrain from providing alternative uses that contain the letter ‘c.”' (Rauscher et al., 1996). Aligned with past work, this manipulation was expected to amplify working memory load and disrupt lexical access (Rauscher et al., 1996), thereby either impeding co-speech gesture production, or adding to the task demands of meaningless and restricted movement prescriptions. Experimenters circled responses that contained a “c” to denote participant failures to comply with task rules. If participants generated several ideas that contained the letter “c,” the experimenter reminded them to please “Please refrain from providing alternative uses that contain the letter ‘c.”' Ideas containing the letter “c” were excluded from analysis, because inclusion of task failures would artificially inflate performance. The experimenter offered an example (e.g., “For the item “box,” an unacceptable alternative use would be “to use as a cup or a decoration, because these ideas contain the letter “c”).

#### 2.2.4 AUT scoring

Fluency was scored as the total number of ideas generated for each of the three AUT stimuli, for each participant. Flexibility was scored as the number of categories generated for each stimulus item. Inter-rater reliability was established by having 20% of the AUT tasks selected randomly (*n* = 90 AUTs) and coded blind by a second independent coder for number of categories. Interrater reliability was excellent, ICC = 0.962, 95% CI (0.943, 0.975). Originality was scored in accordance with the Subjective Scoring Method (Silvia, 2011; Silvia and Benedek, 2019), which was accomplished via rater evaluations of participants' creativity. For the present experiment, two raters, blind to participants' group assignments, independently provided subjective ratings (spanning a 0–5 scale) of each idea, then calculated three average ratings for each participant's three AUT trials. For example, if a participant generated three ideas during baseline, with ratings of 3, 4, and 5, the average subjective originality for that trial would be 12/3 = 4. Three primary criteria were considered when assigning ratings: (1) uncommonness, or uniqueness/infrequency of an idea compared to the entire sample, (2) remoteness, or distance from the intended use of the stimulus item (e.g., writing with a pencil), and (3) cleverness, or perceived humor, irony, insight, and intelligence of each idea (Silvia and Benedek, 2019). Ideas were awarded higher ratings when they were uncommon, remote, and clever, whereas ideas that ranked high on one or two factors, but not all three, tended to receive lower ratings. For example, using a key as a weapon is a remote idea, but was commonly generated within the total sample, and was generally not considered clever, unless more detail or context was offered by the participant. Interrater reliability was good for the item pencil ICC = 0.857, 95% CI (0.808, 0.894), and good to excellent for the items shoe ICC = 0.898, 95% CI (0.862, 0.925) and key ICC = 0.905, 95% CI (0.871, 0.930). Originality was also scored using statistical infrequency (i.e., point-based tally of unique responses, expressed as infrequent ideas generated by less than, or equal to, 10 and 20% of the ideational pool; Plucker et al., 2014). Specifically, each participant received two points for ideas that were expressed by ≤ 10% of the sample (*n* = 15), one point for ideas that were expressed by ≤ 20% of the sample (*n* = 30), and zero points for ideas expressed by ≥21% of the sample to render a composite total score for each participant.

### 2.3 Gesture coding

Videos were coded for 150 participants (a video camera malfunction prevented recording all three AUT trials for one participant assigned to the meaningful movement group). Participants' total gesture frequency was measured by coding all iconic gestures, which are gestures “that in form and manner of execution exhibit a meaning relevant to the simultaneously expressed linguistic meaning” (McNeill, 1992, p. 354; Kirk and Lewis, 2017). Gestures were defined as movement that conveys semantic meaning, such as using one's hands to illustrate securing hair with a pencil. To code total gesture frequency, gestures were identified and counted from video recording if they belonged to at least one of four categories (see Table 2). Total gesture frequency was established by summing the number of gestures depicted within the four categories. In some instances, multiple categories were recorded but were marked as belonging to one distinct gesture, so as not to artificially inflate the total number of gestures performed, given that our primary research question was to establish how total gesture frequency impacts creative thinking. To establish inter-rater reliability, 20% of the video segments permitting gesture were selected randomly (*n* = 50 videos) and coded blind by a second independent coder for total gestures. Interrater reliability was excellent, ICC = 0.956, 95% CI (0.924, 0.975).

**Table 2:** Categories used to code gesture.

**Iconic gesture type**	**Description**	**Example**
Target item manipulation	Actions performed on AUT stimuli	Digging with a key
Spatial	Conveys shape, size, or movement path	Spacing hands far apart to signify a large shoe
Body part as object	Hands represent an object	Using fingers as drumsticks
Observer viewpoint	Actions depict a third-person perspective	Using fingers to mimic a person running
Other iconic	Conveys extraneous/unrelated semantic information	Throwing hands in the air while saying, “I have no idea!”

Participants often depicted multiple categories within the same gesture, such as acting as if to use a pencil as a mustache (target item manipulation), with the finger as a pencil (body part as object), or miming the size of a big shoe (spatial) to be used as a hat (target item manipulation). All possible categories were coded but were scored as only one distinct gesture.

## 3 Statistical analyses and results

### 3.1 Divergent thinking performance

Baseline AUT performance characteristics are shown in Table 3. Baseline bivariate correlations between divergent thinking outcomes and gesture are presented in Table 4.

**Table 3:** Baseline performance characteristics of outcome variables.

**Variable**	**M**	**SE**	***R* (min–max)**	**Skew**	**Kurtosis**
Fluency	10.5	0.322	3–20	0.369	−0.402
Flexibility	6.69	0.245	1–15	0.143	−0.055
Originality (Rarity)	9.37	0.517	0–31	0.504	0.100
Originality (subjective)	2.23	0.053	0.85–3.86	−0.586	−0.520

**Table 4:** Baseline correlations between outcomes and gesture.

**Variable**	**1**	**2**	**3**	**4**	**5**
1. Fluency	1	0.613^**^	0.555^**^	−0.032	0.210^*^
2. Flexibility		1	0.801^**^	0.493^**^	0.248^**^
3. Originality (rarity)			1	0.640^**^	0.352^**^
4. Originality (subjective)				1	0.219^**^
5. Baseline gesture					1

^**^Correlations are significant at p < 0.01.

^*^Correlations are significant at p < 0.05.

Using linear mixed model analysis, we evaluated the impact of a movement manipulation (between-subjects factor; meaningful, meaningless, and restricted movement groups) on creativity across the AUT trial structure (within-subjects factor; baseline, movement manipulation, movement manipulation + cognitive load). The dependent variable was AUT performance [i.e., four separate models for fluency, flexibility, originality (rarity method), and originality (subjective method)]. For all analyses, statistical significance was set at an alpha level of 0.05. We examined the interaction between movement manipulation group × AUT trial structure, which demonstrated whether divergent thinking performance across the trial structure differed between the movement manipulation groups. In the case of a significant interaction (*p* < 0.05).

#### 3.1.1 AUT fluency

For the first linear mixed model, results indicated that there was no significant interaction between the movement manipulation groups and AUT trial structure regarding fluency, *F*_(2, 296)_ = 0.55, *p* = 0.699, η*p*^2^ = 0.007. There was a significant decrease in fluency across the trial structure, *F*_(2, 296)_ = 52.019, *p* < 0.001, η*p*^2^ = 0.260. On average, participants generated more ideas during baseline than they did during the movement manipulation trial (*p* = 0.039, 95% CI = 0.026, 1.037) and the movement manipulation + cognitive load trial (*p* < 0.001, 95% CI = 2.043, 3.174). On average, participants also generated more ideas during the movement manipulation trial than they did during the movement manipulation + cognitive load trial (*p* < 0.001, 95% CI = 1.547, 2.607). Additionally, there were no statistically significant differences in fluency between movement manipulation groups, *F*_(2, 148)_ = 0.052, *p* = 0.949, η*p*^2^ = 0.001.

#### 3.1.2 AUT flexibility

Results for the second model indicated that there was no significant interaction between the movement manipulation groups and AUT trial structure regarding flexibility, *F*_(2, 296)_ = 0.911, *p* = 0.458, η*p*^2^ = 0.012. There was a statistically significant decline in flexibility across the trial structure, *F*_(2, 296)_ = 24.45, *p* < 0.001, η*p*^2^ = 0.142. On average, participants generated ideas across a greater number of diverse categories during the baseline trial than they did during the movement + cognitive load trial (*p* < 0.001, 95% CI = 0.798, 1.672). Participants also generated ideas across a greater number of diverse categories during the movement manipulation trial than they did during the movement manipulation + cognitive load trial (*p* < 0.001, 95% CI = 1.016, 1.828). The mean difference in flexibility between baseline and the movement manipulation trial was not significant (*p* = 0.428, 95% CI = .652, 0.278). Additionally, there were no statistically significant differences in flexibility between movement manipulation groups, *F*_(2, 148)_ = 1.3, *p* = 0.276, η*p*^2^ = 0.017.

#### 3.1.3 AUT originality

Results for the third model indicated that there was no significant interaction between the movement manipulation groups and AUT trial structure regarding originality (rarity), *F*_(2, 296)_ = 0.565, *p* = 0.688, η*p*^2^ = 0.008. There was a statistically significant decline in originality across the trial structure, *F*_(2, 296)_ = 19.699, *p* < 0.001, η*p*^2^ = 0.117. On average, participants generated a greater number of rare ideas during the baseline trial than they did during the movement manipulation + cognitive load trial (*p* < 0.001, 95% CI = 1.360, 3.297). Participants also generated a greater number of rare ideas during the movement manipulation trial than they did during the movement manipulation + cognitive load trial (*p* < 0.001, 95% CI = 1.840, 3.494). The difference between baseline and the movement manipulation trial was not statistically significant (*p* = 0.478, 95% CI = −1.280, 0.602). Additionally, there were no statistically significant differences in originality between movement manipulation groups, *F*_(2, 148)_ = 2.691, *p* = 0.071, η*p*^2^ = 0.035. Results for the fourth model indicated that there was no significant interaction between the movement manipulation groups and AUT trial structure regarding originality (subjective), *F*_(2, 296)_ = 1.509, *p* =0.201, η*p*^2^ = 0.020. There were also no statistically significant differences in originality across the trial structure, *F*_(2, 296)_ = 2.522, *p* = 0.082, η*p*^2^ = 0.017. There was a statistically significant difference in originality between movement manipulation groups *F*_(2, 148)_ = 3.159, *p* = 0.045, η*p*^2^ = 0.041. On average, participants in the meaningful movement group generated more original ideas than those in the meaningless movement group (*p* = 0.030, 95% CI = 0.024, 0.453). On average, participants in the restricted movement group generated more original ideas than those in the meaningless movement group (*p* =0.032, 95% CI = 0.021, 0.452). The difference between the meaningful and restricted movement groups was not significant (*p* = 0.986, 95% CI = −0.213, 0.217).^1^

### 3.2 Exploratory analyses

Baseline gesture frequency was positively correlated with all four creativity outcome measures: fluency, *r*_(146)_ = 0.210, *p* = 0.01; flexibility, *r*_(146)_ = 0.248, *p* = 0.002; objective originality, *r*_(146)_ = 0.352, *p* < 0.001; subjective originality, *r*_(146)_ = 0.219, *p* = 0.007. Therefore, we explored whether individual differences in baseline gesture frequency moderated the effects of an embodied manipulation on AUT performance across the trial structure (Hayes, 2017; PROCESS v.3.0). Regression models were constructed by specifying the change in AUT performance as a continuous outcome variable.

Specifically, change scores compared the movement manipulation trial to baseline to represent how a movement manipulation impacted AUT scores relative to baseline (e.g., negative change scores would indicate that the movement manipulation decreased performance relative to the baseline trial). Change scores also compared the movement manipulation + cognitive load trial to the movement manipulation trial to represent how a movement manipulation with cognitive load impacted AUT scores relative to a movement manipulation only (e.g., negative change scores would indicate that the amplification of cognitive load decreased performance relative to the movement manipulation trial). Lastly, change scores compared the movement manipulation + cognitive load trial to baseline to represent how a movement manipulation with cognitive load impacted AUT scores relative to baseline (e.g., negative change scores would indicate that the amplification of cognitive load decreased performance relative to the baseline trial). Additionally, movement manipulation group was specified as a multicategorical predictor variable and gesture was specified as a continuous moderator variable in each regression model. Table 5 summarizes the effects of the moderation analyses.

**Table 5:** Summarization of the effects of exploratory moderation analyses.

**Divergent thinking performance difference**	**Outcomes**			
	**Fluency**	**Flexibility**	**Originality (rarity)**	**Originality (subjective)**
Movement manipulation – baseline	X	X	X	X
Movement manipulation + cognitive load – movement manipulation	X	X	✓^a^	✓^b^
Movement manipulation + cognitive load – baseline	X	X	✓^a^	✓^b^

X = no evidence (✓= evidence) for gesture moderating the effects of a movement manipulation on changes in divergent thinking performance.

^a^Infrequent gesturers were more negatively impacted by a meaningful movement manipulation.

^b^Frequent gesturers were more positively impacted by a meaningful movement manipulation.

#### 3.2.1 Changes in fluency and flexibility

Evidence for moderation was not found for any movement manipulation group-level changes in fluency or flexibility (*p*s > 0.08). Evidence for moderation was not found for any movement manipulation group-level changes in originality (rarity), Δ*R*^2^ = 0.032, *F*_(5, 142)_ = 0.927, *p* = 0.465, from the movement manipulation trial to baseline.

#### 3.2.2 Changes in originality (rarity)

**Figure 2:**
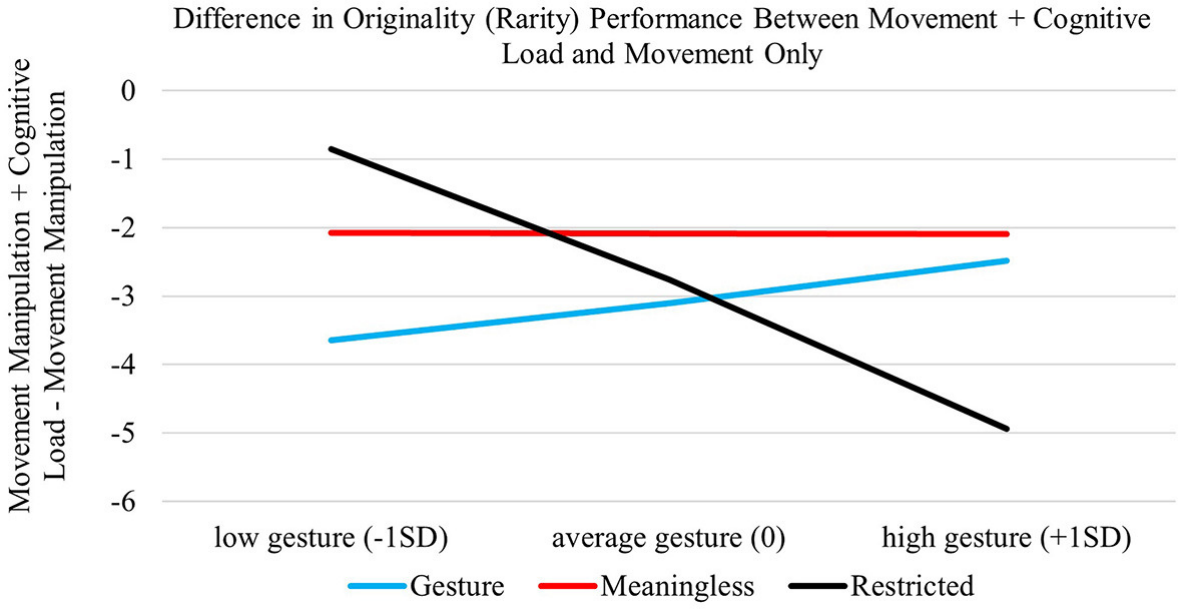
Baseline gesture moderated the effects of a movement manipulation on changes in originality (rarity) performance between increased cognitive load and movement only.

Evidence for moderation was found for movement manipulation group-level changes in originality (rarity), Δ*R*^2^ = 0.107, *F*_(5, 142)_ = 3.39, *p* = 0.006, from the movement manipulation + cognitive load trial to the movement manipulation trial. Specifically, baseline gesture frequency appeared to moderate the effects of restricted movement compared to meaningful gesture on the change in originality (rarity), β = −0.78, 95% CI = −1.297, −0.263, SE = 0.261, *p* = 0.003, as well as the effects of restricted compared to meaningless movement on the change in originality (rarity), β = −0.605, 95% CI = −1.203, −0.006, SE = 0.303, *p* = 0.048. Baseline gesture frequency did not moderate the effects of meaningless movement compared to meaningful gesture on the change in originality (rarity), β = −0.175, CI = −0.833, 0.482, SE = 0.332, *p* = 0.599. These effects are illustrated in Figure 2. To probe the significant interactions, we compared the effects of the movement manipulation groups on the change in originality (rarity) across three different levels of the moderator. Infrequent baseline gesturers (−1 SD from the mean) demonstrated higher originality (rarity) when given cognitive load in the restricted movement group compared to when they were in the meaningful gesture group, β = 2.798, 95% CI = 0.247–5.349, SE = 1.290, *p* = 0.032. No other comparisons were statistically significant.

**Figure 3:**
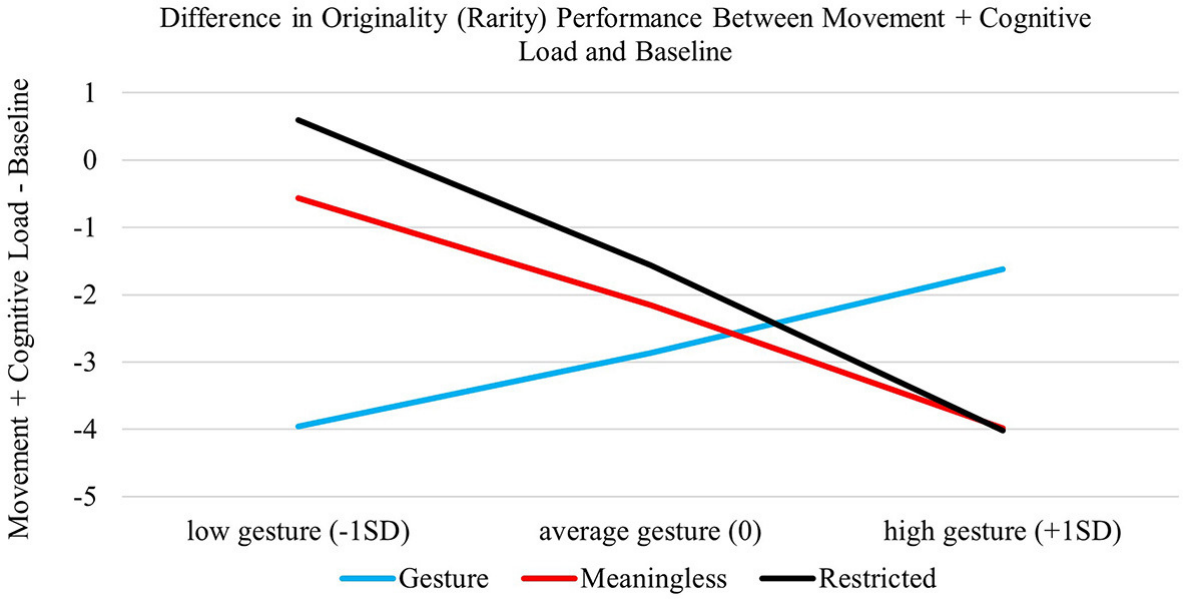
Baseline gesture moderated the effects of a movement manipulation on changes in originality (rarity) performance between increased cognitive load and baseline.

Evidence for moderation was also found for movement manipulation group-level changes in originality (rarity), Δ*R*^2^ = 0.127, *F*_(5, 142)_ = 4.14, *p* = 0.002, from movement manipulation + cognitive load to baseline. Specifically, the interaction terms indicated that baseline gesture frequency moderated the effects of restricted movement compared to meaningful gesture on the change in originality (rarity), β = −1.032, 95% CI = −1.622, −0.443, SE = 0.298, *p* = 0.001, as well as the effects of meaningless movement compared to meaningful gesture on the change in originality (rarity), β = −0.855, CI = −1.604, −0.105, SE = 0.379, *p* = 0.026. Baseline gesture frequency did not moderate the effects of restricted movement compared to meaningless movement on the change in originality (rarity), β = −0.178, 95% CI = −0.861, 0.505, SE = 0.345, *p* = 0.608. These effects are illustrated in Figure 3.

To probe the significant interactions, we compared the effect of the movement manipulation groups on the change in originality (rarity) across three different levels of the moderator. Infrequent baseline gesturers (−1 SD from the mean) experienced higher originality (rarity) when given a cognitive load plus movement (compared to baseline) in the restricted movement group compared to meaningful gesture, β = 4.554, 95% CI = 1.644, 7.464, SE = 1.472, *p* = 0.002. Infrequent baseline gesturers also experienced higher originality (rarity) when given a cognitive load plus movement (compared to baseline) in the meaningless movement group compared to meaningful gesture, β = 3.398, CI = 1.644, 7.464, SE = 1.472, *p* = 0.032. No other comparisons were statistically significant.

#### 3.2.3 Changes in originality (subjective)

Evidence for moderation was not found for any movement manipulation group-level changes in originality (subjective), Δ*R*^2^ = 0.033, *F*_(5, 142)_ = 0.953, *p* = 0.449, from movement manipulation to baseline. However, evidence for moderation was found for movement manipulation group-level changes in originality (subjective) for subsequent comparisons.

**Figure 4:**
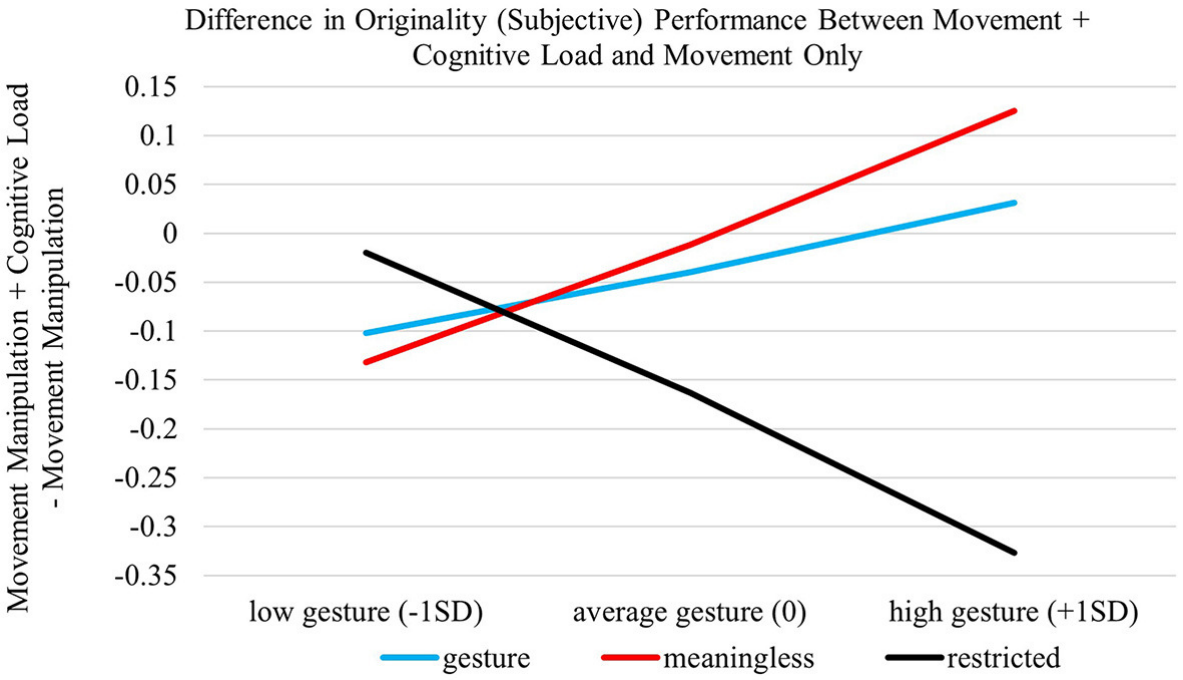
Baseline gesture moderated the effects of a movement manipulation on changes in originality (subjective) performance between increased cognitive load and movement only.

Evidence for moderation was found for movement manipulation group-level changes in originality (subjective) Δ*R*^2^ = 0.110, *F*_(5, 142)_ = 3.496, *p* = 0.005, from the movement manipulation + cognitive load trial to the movement manipulation trial. Specifically, the interaction terms indicated that baseline gesture frequency moderated the effects of restricted movement compared to meaningful gesture on the change in originality (subjective), β = −0.065, 95% CI = −0.113, −0.018, SE = 0.024, *p* = 0.007, as well as the effects of restricted compared to meaningless movement on the change in originality (subjective), β = −0.084, 95% CI = −0.139, −0.029, SE = 0.028, *p* = 0.003. However, baseline gesture frequency did not moderate the effects of meaningless movement compared to meaningful gesture on the change in originality (subjective), β = 0.018, 95% CI = −0.042, 0.079, SE = 0.030, *p* = 0.548. These effects are illustrated in Figure 4.

To probe the aforementioned significant interactions, we compared the effect of the movement manipulation groups on the change in originality (subjective) across three different levels of the moderator. Frequent baseline gesturers (+1 SD from the mean) experienced lower originality (subjective) when given a cognitive load plus movement (compared to a movement manipulation alone) in the restricted movement group relative to both meaningful gesture, β = −0.358, 95% CI = −0.603, −0.114, SE = 0.124, *p* = 0.004 and meaningless movement groups, β = −0.452, 95% CI = −0.723, −0.181, SE = 0.137, *p* = 0.001. No other comparisons were statistically significant.

**Figure 5:**
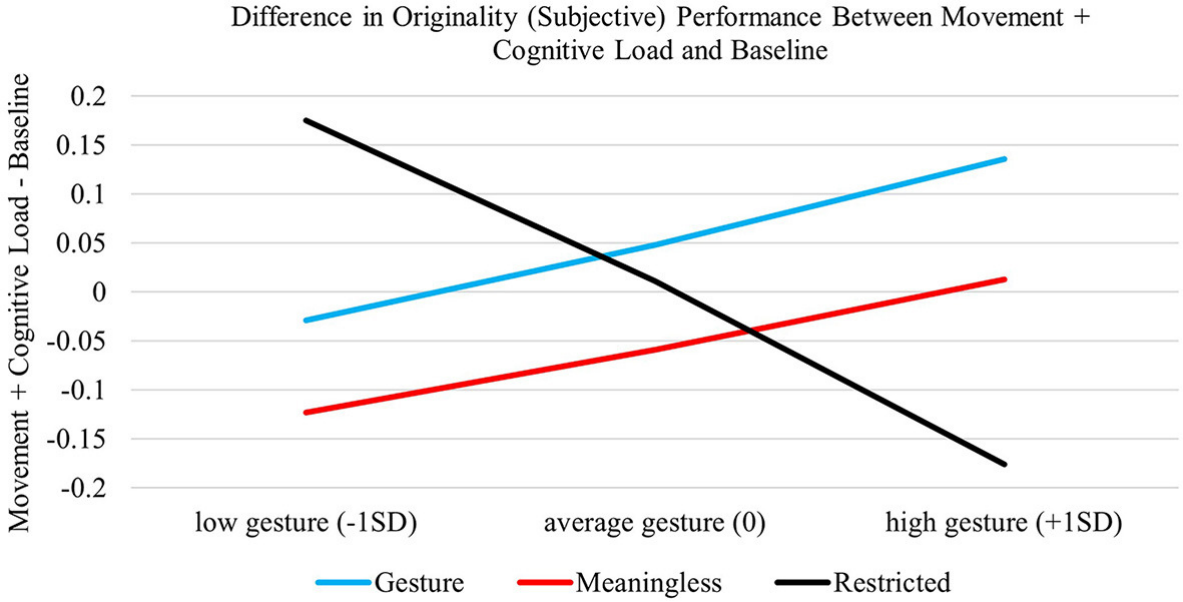
Baseline gesture moderated the effects of a movement manipulation on changes in originality (subjective) performance between increased cognitive load and baseline.

Evidence for moderation was also found for group-level changes in originality (subjective), Δ*R*^2^ = 0.073, *F*_(5, 142)_ = 2.24, *p* = 0.054, from movement manipulation + cognitive load to baseline. Given the exploratory nature of this analysis, I followed up this non-significant *p*-value by interpreting interactions. Specifically, the interaction terms indicated that baseline gesture frequency moderated the effects of restricted movement compared to meaningful gesture on the change in originality (subjective), β = −0.077, 95% CI = −0.135, −0.018, SE = 0.029, *p* = 0.01, as well as the effects of restricted movement compared to meaningless movement on the change in originality (subjective), β = −0.072, 95% CI = −0.140, −0.005, *p* = 0.036. Baseline gesture frequency did not moderate the effects of meaningless movement compared to meaningful gesture on the change in originality (subjective), β = −0.004, 95% CI = −0.078, −0.07, SE = 0.037, *p* = 0.908. These effects are illustrated in Figure 5.

To probe the significant interactions, we compared the effect of the movement manipulation groups on the change in originality (subjective) across three different levels of the moderator. Frequent baseline gesturers (+1 SD from the mean) experienced lower originality (subjective) when given a cognitive load plus movement (compared to baseline) in the restricted movement group compared to meaningful gesture, β = −0.312, CI = −0.613, −0.012, SE = 0.152, *p* = 0.042. No other comparisons were statistically significant.

## 4 Discussion

The present research study examined whether meaningful movement (i.e., gesture) performed during three divergent thinking tasks (i.e., AUTs) impacted creative divergent thinking. While experimental work in embodied cognition purports that movement is a beneficial tool for mitigating cognitive workload during effortful tasks, this project demonstrates that the role of movement for creative thinking may be more nuanced. Specifically, we found that a meaningful movement manipulation did not differentially influence divergent thinking across the trial structure, compared to meaningless movement and restricted movement. Additionally, and in contrast with a COT perspective, gesture frequency decreased in the meaningful movement group when cognitive load was induced. However, differences in individuals' baseline gesture frequency were shown to moderate the effect of an embodied manipulation on divergent originality. Taken together, these results provide new insight into the nature of embodiment-creative cognition relationships by revealing that individual differences in movement propensity paired with cognitive effort involved in the task may help inform the utility of embodied manipulations for creative divergent thinking.

Most embodied creativity research has not evaluated changes in creative performance across a progressively challenging task structure, employed comparative conditions that are designed to disrupt creative thinking (i.e., meaningless movement), or included adequate non-movement comparison groups (see Frith et al., 2019). Results indicated that fluency declined across the trial structure for all movement manipulation groups. More specifically, cognitive load impaired divergent thinking to a similar extent between meaningful, meaningless and restricted movement manipulation groups. That is, flexibility and originality (rarity) performances worsened when cognitive load was added by asking participants to refrain from providing ideas that contain the letter “c” (e.g., sketch or color for the pencil stimulus, Rauscher et al., 1996). Only subjective originality was maintained for all movement manipulation groups across the trial structure. These outcomes were unexpected, as we predicted that only restricted and meaningless movement groups would experience performance impairments in divergent thinking. This is because the restricted manipulation was designed to hinder performance by preventing embodied offloading and the ability to effectively share mental resources with the task environment. The meaningless movement manipulation was also hypothesized to prevent embodied offloading and was expected to yield the greatest decline in performance across the trial structure as repetitive, task-irrelevant movement has been shown to tax working memory resources and to be maximally distracting during problem-solving (Cook et al., 2012). Although examining the effects of a movement manipulation in isolation may suggest that meaningful movement does not impact divergent thinking outcomes; it is likely that the relationship between meaningful gesture and divergent thinking is more nuanced with several possible conclusions.

First, results appear to suggest that meaningful gesture may actually induce cognitive load in certain situations. This seems to conflict with research that has explored the function of gesture as lexical movement which accompanies and represents speech content (Hadar, 1989; McNeill, 1992; Rauscher et al., 1996). Specifically, early work argues that restricting such lexical movement (i.e., co-speech hand gesture) causes speech disfluency during the explanation of video clips (Rauscher et al., 1996). However, restricting movement (or prescribing meaningless movement) in the present study did not differentially impact speech content when compared to meaningful gesture, which was expected to encourage the mental representation, and verbal communication, of novel ideas. These discrepant outcomes may be due to several factors. For example, Rauscher et al. (1996) asked participants to gesture while verbally explaining 3-min-long cartoon clips, which may be easier to recollect than the process of effortfully generating creative ideas. To this end, gesture may impact divergent thinking differently than speech fluency during explanatory tasks, as divergent thinking incorporates the challenge of producing fluent, flexible, and original responses. Additionally, the cognitive load imposed in the present experiment was the same load manipulation used by Rauscher et al. (1996), which required verbalization of responses that omit the letter “c.” Therefore, for some individuals, this added cognitive load may have interacted with task demands unique to divergent thinking, thereby negating the predicted favorable effects of embodied offloading.

Examining individual differences in gesture proclivity also allows for an alternate explanation of the present findings—namely that the impact of gesture on divergent thinking may depend, in part, on individuals' natural tendencies to use movement or to remain still while solving problems. More specifically, in the present study, we found that infrequent baseline gesturers (−1 SD from the mean) achieved better originality (rarity) performance under cognitive load when asked to engage in restricted movement. Although unexpected, perhaps this is because infrequent gesturing at baseline, followed by forced production of meaningful gestures taxed cognitive resources to a greater extent than being asked to remain completely still or to merely produce unchanging, repetitive hand circles. This suggests that a limited movement manipulation may have better aligned with infrequent gesturers' natural movement preferences (or predispositions), but that meaningful movement conflicted with their ability to successfully engage in effortful creative divergent thinking.

Additionally, individuals who gestured infrequently at baseline may also have been more negatively impacted by the pairing of a task-relevant, movement manipulation with increased cognitive load. As aforementioned, divergent thinking tasks are potentially more effortful than verbal explanation tasks assessed in prior embodied cognition work (Rauscher et al., 1996). Therefore, it is conceivable that an added manipulation requiring task-relevant gesture production, while generating novel ideas, augmented working memory constraints among people who were less likely to naturally rely on gesture. In other words, lexical constraints may have been further amplified when a movement manipulation was coupled with cognitive load because participants were required to simultaneously remember to gesture while constraining their speech. This may have intensified cognitive load to an extent that rendered physical offloading ineffectual.

On the other hand, gesture may have attenuated cognitive load for some participants. In addition to the maintenance of subjective originality performance across the trial structure, frequent baseline gesturers (+1 SD from the mean) experienced lower originality (subjective) performance under cognitive load when asked to engage in restricted movement relative to meaningful movement. Perhaps this is because frequent gesturing at baseline, followed by a restricted movement manipulation was misaligned with participants' natural gesture tendencies. This movement incompatibility across the remainder of the trial structure may have taxed cognitive resources to a greater extent than being asked to move via gesture or via repetitive hand circles. This also supports the integrated cognitive offloading and EF approach, which suggests that task performance is a function of cognitive resource demand and offloading accessibility. That is, divergent thinking may be facilitated during challenging tasks when there are opportunities for embodied offloading to preserve executive resources (Kirsh and Maglio, 1994; Scaife and Rogers, 1996; Wilson, 2002; Martin and Schwartz, 2005; Pouw et al., 2014; Risko et al., 2014; Risko and Gilbert, 2016). Although, it is likely that cognitive demands differed between individuals, which may further clarify why gesturing frequently at baseline benefited some people, but not others.

Another reason for why baseline gesture inclinations moderated divergent originality in this sample may be that individuals differ in visuo-spatial abilities that map onto motor imagery. The finding that frequent baseline gesturers' subjective originality performance appeared to be harmed when their movements were restricted may be explained by work illustrating that lower visuo-spatial ability lends itself to increased engagement in task-relevant movement (Beilock and Goldin-Meadow, 2010). Although we did not assess visuo-spatial ability in the present study, previous research has shown that individuals with low spatial rotation abilities tend to gesture more when describing their mental transformation strategies than individuals with higher abilities (Göksun et al., 2013). Spatial rotation tasks require mentally representing different orientations of two- or three-dimensional objects, in order to match the shape of an object with a target item that is presented at a different angle of rotation (Berger et al., 1990). Additionally, Chu et al. (2014) found that the frequency of representational gestures accompanying speech were negatively associated with mental conceptualization, visuo-spatial working memory, and spatial rotation abilities.

Some evidence also points to greater reliance on spontaneous co-speech hand gestures when the spatial visualization of task elements is difficult (Alibali et al., 2000; Hostetter et al., 2007; Melinger and Kita, 2007; Kita and Davies, 2009; Chu and Kita, 2011) and cannot be accomplished via internal thought processes alone (Chu and Kita, 2011). Tasks that require visuo-spatial information processing, such as asking people to describe how to wrap a gift, have been shown to stimulate motor imagery and may incite more gestures compared to tasks that evoke predominantly visual imagery (e.g., describing a painting), or tasks that integrate abstract concepts (e.g., providing opinions on the representation of women in politics, Feyereisen and Havard, 1999). This is because visual and abstract imagery orientations do not rely as heavily on planned motor responses (Feyereisen and Havard, 1999), whereas visuo-spatial processing is thought to stimulate both imagined and overt physical actions capable of satisfying task-relevant goals (Wesp et al., 2001; Miller and Franz, 2005; Trafton et al., 2006; Hostetter and Alibali, 2007, 2008). AUT instructions require participants to generate imagined physical uses for common objects (e.g., bend an old key to use as a hook for clothing). This process may have stimulated motor imagery and provoked co-speech gestures to reinforce divergent thinking performance among frequent baseline gesturers, but infrequent baseline gestures may have been better able to rely upon motor imagery alone. However, future research is needed to explore this speculation, as visuo-spatial ability is not a unitary construct and is therefore open to a much broader range of experimental manipulations.

Taken together, perhaps individuals who gestured more at baseline experienced greater difficulties constructing strong mental representations of imagined uses for stimulus items, so gesturing was used as a way to help represent the task and guide idea generation. If high baseline gesturers were then prescribed restricted movement, a decline in subjective originality performance would not be surprising, as meaningful movement was no longer an option for promoting effective task representation. In contrast, imagined mental representations of task elements may have been more easily constructed among those who did not gesture often at baseline, potentially reflecting higher visuo-spatial skill; therefore, as gesturing was not essential, a meaningful movement assignment may have been distracting and detrimental to changes in AUT originality performance. Because visuo-spatial skill and cognitive load may differentially affect individuals' ability to effectively construct representational mental images and produce task-specific gestures in conjunction with speech, continued work should begin to experimentally investigate cognitive explanations for why creative divergent thinking may elicit more gestures for some individuals than for others.

Given that reliance on gesture at baseline emerged as a moderator of changes in divergent originality, it may be beneficial to argue that movement is a contextual affordance for creativity when it supports an individual's cognitive abilities, motivations, and natural inclinations to engage in movement. A greater understanding of this “person-context fit” may help researchers further investigate ways to enhance creative thinking and extend findings to applied settings. For example, from a developmental perspective, children tend to use representational movement in place of language because their motor repertoire is more proficient than their verbal abilities (see e.g., Frith et al., 2019). Some adults may not need to rely on movement as a way to effectively communicate their mental states, and if gesture is not *needed* to meaningfully convey ideas and reduce cognitive workload, then being forced to use it may diminish the quality of creative thought and behavior. Therefore, it is possible that, in certain situations, forced offloading introduces a potential confound in the movement-creativity relationship. Additionally, and although speculative, given the social function of gesture (Church et al., 2007), experimenters may consider instructing participants to explain their ideas in a more collaborative manner. Recall that we implemented the following standard task instructions for the meaningful movement group: “Please be sure to gesture with your hands while thinking of ideas for alternative uses,” which may have been less effective for socially motivated individuals than, for example, modifying instructions to prompt sharing knowledge with another person. Future work should examine the role of individual differences in gesture on creative thinking in divergent thinking tasks with a collaborative component (e.g., “Please be sure to gesture with your hands while *sharing and describing* your ideas for alternative uses *with me*.”). It can be argued that, in the present study, mental effort was largely internally-oriented toward generating creative responses, but it is worth exploring whether individuals may be more apt to gesture when effort is more externally-oriented to share ideas with another person, and how this relates to natural inclinations to use gesture at baseline.

Because two well-established originality metrics were impacted by individual differences in meaningful movement, the findings of this experiment are particularly informative, compared to the use of a single measure alone. No embodied creativity experiment has integrated measurements of both the rarity (statistical infrequency) and perceived value (external ratings) of original divergent responses, despite recommendations to report both objective and subjective scoring techniques to provide more comprehensive, reliable, and valid indices of the uncommonness and appropriateness of ideas (Silvia et al., 2008). In addition, no embodied creativity experiment has explored the role of individual differences in gesture on creative cognition. This experiment offers a substantial contribution to the literature because probing individual differences in embodied creativity may lead to a better understanding of how movement may, or may not, help people think of creative solutions to everyday problems. By continuing to explore individual differences in task-relevant embodied actions, a more person-centered approach to disentangling the cognitive basis of creative divergent thinking may help inform evidence-based strategies for supporting creativity in educational and professional development, as well as personal achievements.

Some limitations of this research are related to the moderation outcomes observed. First, we explored several models, with many demonstrating no significance of gesture as a moderator of the change in divergent thinking performance. Second, it is important to note that making strong conclusions from moderation outcomes may be premature, as the confidence intervals spanning moderation effects were often imprecise. Another limitation of this study is that the outcomes observed are representative of a single research visit in an isolated laboratory context; therefore, conclusions about the enduring or contextual role of gesture in creative divergent thinking cannot be formed on the basis of this experiment. Considering this factor in addition to our moderation analyses, future research should examine individual differences in embodiment as a focal outcome and implement within-subject designs that assess individuals across multiple days to disentangle whether embodied predispositions may be specific to an isolated task/laboratory visit, or if they endure across assessment periods.

In conclusion, this study demonstrated that the cognitive processes involved in solving effortful creativity problems may be more sensitive to individual abilities than the movement prescription alone. From this foundation, future research in embodied creative cognition should ask not only what kinds of problems and contexts are influenced by movement, but also for whom is movement useful?

## Data Availability

The raw data supporting the conclusions of this article will be made available by the authors, without undue reservation.

## Footnotes

^1^Participants did not have physical stimuli in front of them during the experiment, as we did not want to constrain gesture production to a specific type of stimuli. However, 24 participants were tested with a pencil on the table during the AUT; Additionally, three participants used the pencil (i.e., physically held the pencil) during the trial requiring idea generation for the stimulus item “pencil;” however, removing these participants/trials from the analyses did not alter the statistical interpretation of any of the models.
